# Mortality Benefits of Antibiotic Computerised Decision Support System: Modifying Effects of Age

**DOI:** 10.1038/srep17346

**Published:** 2015-11-30

**Authors:** Angela L. P. Chow, David C. Lye, Onyebuchi A. Arah

**Affiliations:** 1 Department of Clinical Epidemiology, Institute of Infectious Disease and Epidemiology, Tan Tock Seng Hospital, Singapore; 2 Department of Epidemiology, Fielding School of Public Health, University of California, Los Angeles (UCLA), Los Angeles, United States; 3 Department of Infectious Diseases, Institute of Infectious Disease and Epidemiology, Tan Tock Seng Hospital, Singapore; 4 Yong Loo Lin School of Medicine, National University of Singapore, Singapore; 5 Center for Health Policy Research, University of California, Los Angeles (UCLA), Los Angeles, United States

## Abstract

Antibiotic computerised decision support systems (CDSSs) are shown to improve antibiotic prescribing, but evidence of beneficial patient outcomes is limited. We conducted a prospective cohort study in a 1500-bed tertiary-care hospital in Singapore, to evaluate the effectiveness of the hospital’s antibiotic CDSS on patients’ clinical outcomes, and the modification of these effects by patient factors. To account for clustering, we used multilevel logistic regression models. One-quarter of 1886 eligible inpatients received CDSS-recommended antibiotics. Receipt of antibiotics according to CDSS’s recommendations seemed to halve mortality risk of patients (OR 0.54, 95% CI 0.26–1.10, *P* = 0.09). Patients aged ≤65 years had greater mortality benefit (OR 0.45, 95% CI 0.20–1.00, *P* = 0.05) than patients that were older than 65 (OR 1.28, 95% CI 0.91–1.82, *P* = 0.16). No effect was observed on incidence of *Clostridium difficile* (OR 1.02, 95% CI 0.34–3.01), and multidrug-resistant organism (OR 1.06, 95% CI 0.42–2.71) infections. No increase in infection-related readmission (OR 1.16, 95% CI 0.48–2.79) was found in survivors. Receipt of CDSS-recommended antibiotics reduced mortality risk in patients aged 65 years or younger and did not increase the risk in older patients. Physicians should be informed of the benefits to increase their acceptance of CDSS recommendations.

Antibiotics are among the major developments in modern medicine, saving countless lives over the decades^1^. Antibiotic use in hospitals has increased substantially^2^,^3^. Recent data from the Netherlands showed that antibiotic use has increased by 22% from 2003 to 2010 (2). Approximately 60% of adults admitted to U.S. hospitals received at least one dose of antibiotics during their stay^4^. However, 41–91% of antibiotics prescribed in hospitals are considered inappropriate^5^.

Overuse and misuse of antibiotics have driven the emergence of antimicrobial resistance^6^,^7^, a serious threat to clinical care^8^. Hospital antimicrobial stewardship programmes have been established to facilitate the optimal use of antibiotics^2^,^4^,^9^,^10^,^11^. Furthermore, antibiotic computerised decision support systems (CDSS) have been developed to improve antibiotic decision-making through the accessibility of patient-specific clinical data and antibiotic guidelines, at the point of prescribing^12^,^13^,^14^,^15^,^16^,^17^. Antibiotic CDSSs are particularly useful for antibiotic selection for empirical therapy, as optimal selection is complex when the causative pathogen is unknown^4^,^18^,^19^. Appropriate empirical treatment is crucial for the resolution of infection and the reduction of mortality^4^,^20^.

Antimicrobial stewardship can improve antibiotic prescribing and clinical outcomes in hospital inpatients^2^. Antibiotic CDSSs could further enhance antibiotic prescribing^13^,^21^, but evidence on the benefits of CDSSs on clinical outcomes is limited^22^. While most physicians recognise the emergence of antimicrobial resistance as an important problem, they are primarily concerned with individual patients’ clinical outcomes rather than the risk of resistance in their antibiotic choices^9^. Understanding the clinical benefits of CDSSs is essential to increase physicians’ confidence in and acceptance of recommendations by antibiotic CDSSs.

We conducted a prospective cohort study to evaluate the effectiveness of a tertiary hospital’s in-house antibiotic CDSS, “Antimicrobial Resistance Utilization and Surveillance Control” (ARUSC), on mortality, readmission, incidence of *Clostridium difficile* infection (CDI), and multidrug resistant organism (MDRO) infection, and the modification of these effects by patient factors.

## Methods

### Study setting and population

The study was conducted in Tan Tock Seng Hospital, a 1500-bed tertiary-care academic centre that serves a diverse ethnic, adult medical and surgical population in Singapore. In 2009, the hospital launched its antibiotic CDSS, “ARUSC”, which integrates antimicrobial stewardship with the hospital’s computerised physician order entry system (CPOE) and provides patient-specific evidence-based antibiotic recommendations at the point of prescribing^16^,^23^.

From September 12, 2011, whenever a physician makes an electronic prescription of piperacillin-tazobactam or a carbapenem for an inpatient, the prescription automatically triggers the launch of ARUSC. Piperacillin-tazobactam and carbapenems are antibiotics of last resort for many bacterial infections, particularly those caused by multidrug-resistant pathogens. Hence, it is crucial to ensure the judicious use of these antibiotics.

All patients admitted to the hospital, from October 1, 2011 through September 30, 2012, who were prescribed piperacillin-tazobactam or a carbapenem for empirical therapy and automatically triggered to receive antibiotic recommendations by ARUSC were included in the study. We included only the first prescription for empirical therapy per patient during the study period. Empirical therapy is the initiation of antibiotic treatment prior to the identification of the infection-causing microorganism. Using a rules-based algorithm, ARUSC provides guidance on antibiotic selection and dosing, based on guidelines developed by the hospital’s antimicrobial stewardship committee, which took into account the local epidemiology of infectious diseases, microbiologic resistance patterns, and incorporated evidence-based international guidelines. Inputs from all clinical departments were considered in the development of the guidelines, which were endorsed by the hospital’s medical board. ARUSC recommends the narrowest-spectrum antibiotic appropriate for common organisms responsible for the diagnosed infection, based on the local epidemiology and antibiotic susceptibility patterns, taking into account the patient’s antibiotic allergies and renal function (Supplementary Table S1). The prescribing physician can either accept or reject ARUSC’s antibiotic recommendations.

Prescriptions for prophylactic or definitive therapy were excluded from the study. We chose to focus our study on empirical therapy, as empirical antibiotic prescriptions were the least concordant with antibiotic guidelines^19^. Empirical antibiotics are the first antibiotics received by the patient in an infective episode and the receipt of appropriate empirical antibiotics is a critical determinant of good clinical outcomes^20^.

### Study design

We assembled a prospective observational cohort, starting from the automatically-triggered launch of ARUSC at the point of antibiotic prescribing up to 30 days post-hospital discharge or 180 days post-antibiotic prescription, whichever was later.

### Outcome variables

We selected 30-day all-cause mortality as the primary outcome, since the key benefit of appropriate empirical antibiotic therapy is 30-day survival gain^13^. As secondary outcomes, we assessed the incidence of CDI and MDRO infection (>2 days and <=180 days after antibiotic prescription)^24^. A CDI was defined as concurrent positive results on faecal samples from parallel testing for *C. difficile* toxin and *C. difficile*-specific enzyme glutamate dehydrogenase antigen using the Techlab *C. difficile* Quik Chek Complete test, without a positive test during the preceding 8 weeks (repeat positive tests during this period suggest recurrence rather than incidence)^25^. Whenever there was discordance in the results of the two tests, a confirmatory GeneXpert *C. difficile* polymerase chain reaction test for the presence of *C. difficile* genetic material was carried out. We defined MDRO as a bacterium that is resistant to three or more of five antibiotic classes^26^. Additionally, we evaluated the incidence of 30-day infection-related readmission rates among survivors. Readmission within 30 days of hospital discharge was a proxy for non-resolution of the infection.

### Exposure variable

Patients’ receipt of antibiotics recommended by ARUSC was determined by electronically matching antibiotics prescribed in the institutional CPOE system with those recommended by ARUSC. A patient was classified as having received an ARUSC intervention if the prescribed antibiotics and those recommended by ARUSC matched exactly on the dose, route, and frequency of administration.

### Covariates

Relevant patient characteristics included socio-demographic data (age, gender, ethnicity, resident status, and ward class), co-morbidities, illness severity, admission to an intensive care unit (ICU) at the time of prescribing, prior antibiotic exposure within 180 days and proton pump inhibitor exposure within 90 days preceding current prescription, prior hospitalisation within 90 days preceding current admission, diagnosed infection for current antibiotic therapy, and the time and day of week when the prescription was made.

We dichotomised age to <=65 and >65 years, representing younger and older age groups. Ward class was based on admission to a private or subsidised room, and used as a surrogate measure of the patient’s socioeconomic status. We defined co-morbidities as follows. Diabetes mellitus: a diagnosis of diabetes with or without complications. Cardiovascular disease: coronary artery disease or congestive heart failure. Liver disease: liver disease of any severity. Renal disease: moderate to severe renal disease. Neoplasm: solid malignant tumour, leukaemia, lymphoma, or any metastasis. Central nervous system (CNS) disease: cerebrovascular disease, dementia. Chronic pulmonary disease: chronic obstructive pulmonary disease. Charlson’s co-morbidity index (CCI)^27^ was derived from the hospital discharge database using coding algorithms developed by Quan H *et al.*^28^. CCI was then categorised into <=5 and >5, representing good and poor chronic health status. Illness severity was determined using biochemical markers measured within 7 days of the prescription. We used C-reactive protein >100mg/l and leukocyte count <4 or >12 × 10 ^ 9/l as proxies for severe infection, and serum creatinine >130 μmol/l as proxy for renal impairment^29^. Data were obtained electronically from ARUSC, institutional electronic medical and pharmacy records, and admission and discharge databases.

The prescribing physician was the physician who initiated the empirical antibiotic prescription that led to the automatically-triggered launch of ARUSC. The attending physician was the physician who was primarily responsible for the patient’s clinical care and outcome for the particular hospitalisation episode. Physicians’ characteristics that were collected included the prescribing physician’s seniority, and the attending physician’s ethnicity and clinical specialty. The seniority of the prescribing physician was determined by the physician’s designation. Interns and residents were classified as juniors, and fellows and attending physicians as seniors. Data on the physician’s designation and ethnicity were obtained from the institution’s human resource database and matched to the physician’s identity and clinical specialty data in ARUSC.

### Statistical analysis

First, we used appropriate descriptive statistics to summarise patients’ characteristics, their respective prescribing and attending physicians and clinical specialties, their receipt of antibiotics according to ARUSC’s recommendations, and subsequent clinical outcomes by diagnosed infection. Next, we explored relationships between the receipt of ARUSC-recommended antibiotics, various patients’ and physicians’ characteristics, and each clinical outcome using multilevel logistic regression models with random intercepts. We fitted two types of such models: model 1 involved nesting of patients within their prescribing physicians, and model 2 nested patients within their attending physicians who in turn were nested within their clinical specialties, to account for clustering within prescribing physicians and clustering within attending physicians and clinical specialties respectively. We then constructed multivariable adjusted multilevel logistic regression models, accounting for potential confounding. We included variables decided *a prior* as factors associated with each clinical outcome particularly those based on prior knowledge to be associated with adherence to antibiotic guidelines in general (not specific for antibiotic CDSSs due to limited information on antibiotic CDSSs). Statistical interactions between age, comorbidities, infectious diagnoses, illness severity, and receipt of antibiotics according to ARUSC’s recommendations, were respectively explored and product terms included in the models where appropriate. The percentages of the total outcome variances that could be explained by differences between prescribing physicians, attending physicians, and clinical specialties respectively were computed^30^. To further adjust for potential confounding due to differences in baseline characteristics in patients who received and did not receive ARUSC-recommended antibiotics, we estimated propensity scores from multilevel exposure models on the receipt of antibiotics according to ARUSC’s recommendations^31^,^32^. Doubly robust estimates were obtained by combining propensity scoring with the multivariable adjusted multilevel logistic regressions above. We conducted further sensitivity analyses by excluding patients whose hospital stay was more than 7 days prior to the antibiotic prescription. We used multiple-imputation for measurement error (MIME) correction for adjustment of potential misclassification of CCI based on a validation sub-study of 198 patients that were randomly sampled from the total cohort for whose medical records were manually reviewed by a physician for the presence of comorbidities^33^. Finally, we assessed non-participation and used inverse-probability-of-selection-weighting to adjust for any potential selection bias. All analyses were performed using SAS version 9.3 (SAS Institute Inc, NC).

Ethical approval for the study was obtained from the National Healthcare Group Domain Specific Research Board and UCLA Institutional Review Boards. The study’s methods were carried out in accordance with the approved guidelines. A waiver of informed consent was granted.

## Results

### Patient characteristics

During the one-year study period, a total of 1886 unique inpatients, among 380,800 patient-days, at Tan Tock Seng Hospital were automatically triggered to receive antibiotic recommendations by ARUSC for prescriptions of piperacillin-tazobactam or a carbapenem for empirical therapy.

Pneumonia (community-acquired and healthcare-associated) (64.3%) was the most commonly diagnosed infection, among which patients were the oldest (mean 74.9 years, SD 14.5) (Table 1). Patients with hepatobiliary or intra-abdominal infections (acute cholecystitis and cholangitis, diverticulitis and diverticular abscess, gallbladder empyema, gastroenteritis, hepatobiliary sepsis, liver abscess, pancreatitis, and peritonitis) had the poorest chronic health status and most severe illness, with almost one-fifth having a CCI > 5 (22.5%) and ICU admission (20.4%) respectively. A total of 470 patients received antibiotics according to ARUSC’s recommendations whilst 1416 did not. A much higher proportion of patients treated for pneumonia (33.2%) received ARUSC-recommended antibiotics, compared with patients with sepsis (12.1%) and urinary tract infection (cystitis, pyelonephritis, and perinephric abscess) (7.1%). Patients with a diagnosis of sepsis (primary bloodstream infection with unknown focus) had the highest 30-day all-cause mortality (28.8%), while patients diagnosed with urinary tract infection had the highest incidence of CDI (8.2%). Among survivors of the hospitalisation episode, 11.2% were readmitted for infection-related causes within 30 days.

### 30-Day All-cause Mortality

On univariate analysis, patient factors were similarly associated with 30-day all-cause mortality in both models (Table 2). Age > 65, CCI > 5, pneumonia, sepsis, and ICU admission were positively associated with mortality. The prescribing physician did not contribute to the variation in mortality, while the attending physician (0.4%) and clinical specialty (1.7%) accounted for small variances.

After controlling for potential confounding in the multivariable multilevel models, receipt of antibiotics according to ARUSC’s recommendations was marginally associated with mortality reduction (Model 1: OR 0.54, 95% CI 0.27–1.11; Model 2: OR 0.52, 95% CI 0.26–1.06). Age > 65 (Model 1: OR 1.46, 95% CI 1.06–2.01; Model 2: OR 1.43, 95% CI 1.03–1.98), CCI > 5 (Model 1: OR 1.97, 95% CI 1.44–2.68; Model 2: OR 2.12, 95% CI 1.54–2.92), sepsis (Model 1: OR 3.00, 95% CI 1.58–5.70; Model 2: OR 2.61, 95% CI 1.30–5.26), and ICU admission (Model 1: OR 1.85, 95% CI 1.31–2.61; Model 2: OR 2.25, 95% CI 1.54–3.29) were all positively associated with mortality.

In the propensity score (PS) adjusted multivariable models, the effect of receipt of antibiotics according to ARUSC’s recommendations (Model 1: OR 0.54, 95% CI 0.26–1.10; Model 2: OR 0.52, 95% CI 0.25–1.05) remained, and the effects of CCI > 5 (Model 1: OR 2.00, 95% CI 1.47–2.71; Model 2: OR 2.18, 95% CI 1.59–2.99) and ICU admission (Model 1: OR 1.96, 95% CI 1.40–2.75; Model 2: OR 2.47, 95% CI 1.70–3.58) were enhanced (Table 3). The propensity score was derived from diagnosed infection, time and day of antibiotic prescription, hospitalisation days prior to antibiotics, prior hospitalisation, and prior antibiotics. At the clinical specialty level, patients managed by a medical service were 1.5 times as likely as those managed by a surgical service to die within 30 days of the receipt of antibiotics (OR 1.53, 95% CI 1.02–2.30).

We selected the PS adjusted two-level model (Model 1: prescribing physician, patient) as the final multivariable model, as the model provided the optimal fit overall. Interactions between the receipt of antibiotics according to ARUSC’s recommendations and age, comorbidities, illness severity, and infectious diagnoses were assessed. Age > 65 was found to interact positively with the receipt of antibiotics according to ARUSC’s recommendations (OR 2.32, 95% CI 1.08–4.98), and the product term was included in the final model.

**Figure 1:**
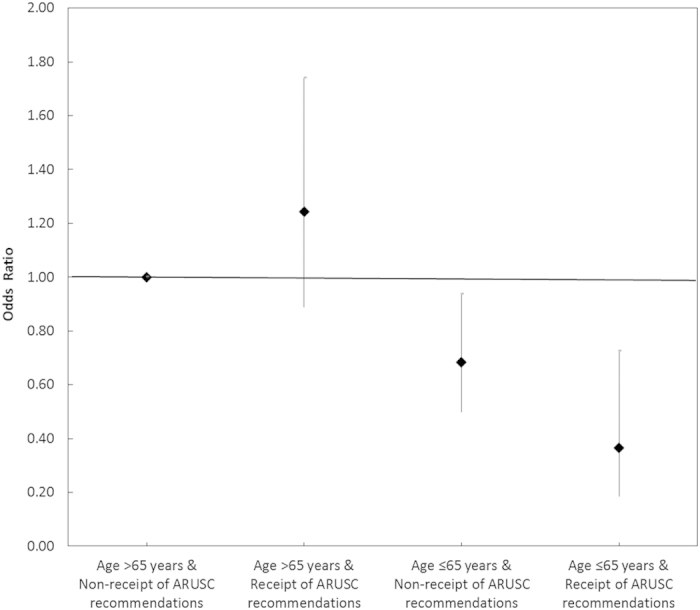
Joint effects of age and receipt of ARUSC recommendations on 30-day all-cause mortality risk.

After adjusting for potential confounding, the receipt of antibiotics according to ARUSC’s recommendations halved the mortality risk of patients (OR 0.54, 95% CI 0.26–1.10, *P* = 0.09) (Table 3). In patients aged 65 and below, the receipt of antibiotics according to ARUSC’s recommendations reduced mortality by 55% (OR 0.45, 95% CI 0.20–1.00, *P* = 0.05). It did not have an effect on mortality in older patients >65 years old (OR 1.28, 95% CI 0.91–1.82, *P* = 0.16) (Table 4). Our study suggests that age (<=65 years) modified the effect of receipt of antibiotics according to ARUSC’s recommendations in reducing mortality risk; as such, the combined effect of age and receipt of antibiotics according to ARUSC’s recommendations was larger than the combination of their component effects (OR 0.37, 95% CI 0.18–0.72, *P* = 0.004) (Fig. 1). Effect estimates for age, receipt of antibiotics according to ARUSC’s recommendations, and interactions did not change notably when we restricted our population to patients who had been hospitalised <= 7 days (data not shown).

### Secondary Outcomes

The multivariable two-level regression including the propensity score showed that ARUSC recommendations had no effect on the subsequent development of CDI (OR 1.02, 95% CI 0.34–3.01, *P* = 0.97) and MDRO infection (OR 1.06, 95% CI 0.42–2.71, *P* = 0.90). After discharge from hospital, patients who received ARUSC-recommended antibiotics did not have an increased rate of 30-day infection-related readmission (OR 1.16, 95% CI 0.48–2.79, *P* = 0.74).

### Sensitivity analysis

With the correction of potential misclassification of CCI, the effect of ARUSC recommendations on 30-day all-cause mortality was unchanged (OR 0.53, 95% CI 0.26–1.10, *P* = 0.09). After adjusting for potential selection bias, the beneficial effect of ARUSC recommendations on 30-day all-cause mortality remained (OR 0.32, 95% CI 0.17–0.63, *P* < 0.01). There was no change in the non-effect on the subsequent development of CDI and MDRO infection, and 30-day infection-related readmission in survivors.

## Discussion

We found that the receipt of antibiotics according to the CDSS’s recommendations reduced the risk for 30-day all-cause mortality in patients aged 65 years and below (OR 0.45, 95% CI 0.20–1.00, *P* = 0.05), and did not increase the risk in older patients >65 years old (OR 1.28, 95% CI 0.91–1.82, *P* = 0.16). A recent meta-analysis on the effect of antimicrobial stewardship interventions intended to increase appropriate antimicrobial therapy for all infections reported no increase in mortality (combined risk ratio 0.91, 95% CI 0.81–1.06, *P* = 0.25)^2^. In the limited studies on the clinical effects of antibiotic CDSSs in hospitals in the US, Europe, and Australia, no difference was observed in 30-day mortality^13^,^34^,^35^. In more recent studies in Germany on ICU patients, low adherence to antibiotic CDSSs’ recommendations was found to be associated with increased risk of ICU mortality in surgical patients (OR 1.56, 95% CI 1.05–2.31, *P* = 0.029)^36^ and patients with sepsis (OR 2.43, 95% CI 1.13–5.24, *P* = 0.02)^37^. Low adherence to CDSSs’ recommendations could be due to physicians’ preference to exercise their own decisions over recommendations by the CDSS when managing complex patients such as those in the ICU^23^,^38^. A cluster-randomised trial including patients in medical wards in an Israeli hospital reported 180-day survival benefit with an antibiotic CDSS (per-protocol analysis: survival was 71% in intervention group vs. 77% in control group, *P* = 0.04)^39^. Our study adds to the body of literature on the benefits of antibiotic CDSSs. CDSSs present a promising future for optimising antibiotic selection and improving clinical outcomes^1^,^40^. More studies are needed in different settings, including Asia^40^. To our knowledge, this study is the first to report on the effects of an antibiotic CDSS in an Asian hospital. Furthermore, none of the previous studies on antibiotic CDSSs has explored the modifying effects of patient factors on clinical outcomes. Our study showed that the joint effect of younger age (<=65 years) and receipt of antibiotics according to CDSS’s recommendations on the reduction of mortality risk was larger than the combination of each of the component effects. Targeted efforts should be made to promote antibiotic CDSSs to physicians managing younger patient populations.

We further observed that receipt of antibiotics according to CDSS’s recommendations had no effect on the subsequent development of CDI and MDRO infection. Although decreases in CDI and MDRO infection have been reported in studies on antibiotic restriction and antimicrobial stewardship policies, the effect of antibiotic CDSSs on such infections have not been studied^2^,^4^,^41^. Previous studies have employed quasi-experimental before-and-after study designs which are prone to ecologic bias. In contrast, our study followed up individual patients longitudinally for the development of CDI and MDRO infection.

Among survivors, patients who received CDSS-recommended antibiotics did not have an increased rate of 30-day infection-related readmission. Other studies have observed an increase in hospital readmissions associated with antimicrobial stewardship interventions intended to decrease excessive prescribing (combined risk ratio 1.26, 95% CI 1.02–1.57, *P* = 0.03), but did not observe a difference in infection-related readmissions between intervention and control groups^2^. However, there has been no published literature on the effect of antibiotic CDSSs on readmissions.

### Strengths and Limitations

Our study has several strengths. First, it followed up a cohort of hospitalised patients longitudinally. The unique patient identifier and admission episode number allowed for electronic linkages across medical and pharmacy records, and administrative databases. As such, all data were electronically collated and any measurement error and misclassification bias was likely to be minimal. Bias analysis revealed that the potential misclassification of CCI had no influence on the outcome. Unlike most studies assessing adherence to antibiotic guidelines which involved study investigators manually reviewing prescriptions that may be error-prone and biased by low inter-rater reliability, our study electronically matched antibiotics prescribed on the CPOE system with ARUSC recommendations to determine patient receipt of antibiotics according to ARUSC’s recommendations. Hence, the exposure measurement was not subject to differential misclassification. Furthermore, we were able to analyse individual patient-level data on their clinical outcomes; hence, our study is not prone to any ecologic bias.

Second, our study used multilevel modelling techniques to account for the clustering of patients within prescribing physicians, and attending physicians and clinical specialties. Many previous studies were not able to do so, and employed standard modelling techniques. The multilevel models provide an improved ability to measure clinical outcomes^22^. Additionally, we were able to study and estimate the relative plausible effects of the prescribing physician, attending physician, and clinical specialty on clinical outcomes.

Third, we derived propensity scores and used doubly robust estimations to compare effects. The corroboration of results from the different methods supported our findings. We further adjusted for potential selection bias in our models and our conclusions remained unchanged.

Our study may have been limited by our inability to study unmeasured patients’ and physicians’ factors, due to the non-availability of those data electronically. However, critical patient factors that could influence clinical outcomes were available and have been included in our models. Prescribing and attending physicians were not found to contribute substantially to the variability in clinical outcomes. Hence, the non-availability of detailed information on physicians is unlikely to bias our results. Our study population did not include children and our findings could not be generalised to paediatric populations. Nonetheless, our findings may be applied to other adult tertiary-care centres with antibiotic CDSSs.

## Conclusion

This study provided insight into the effectiveness of an antibiotic CDSS in an Asian hospital. The receipt of antibiotics according to the CDSS’s recommendations reduced the 30-day all-cause mortality risk in patients aged 65 and below, and did not increase the risk in older patients. Physicians should be informed of the mortality benefits to patients, to increase their acceptance of antibiotic recommendations by CDSSs in their clinical practice.

## Additional Information

**How to cite this article**: Chow, A. L. P. *et al.* Mortality Benefits of Antibiotic Computerised Decision Support System: Modifying Effects of Age. *Sci. Rep.* **5**, 17346; doi: 10.1038/srep17346 (2015).

## Supplementary Material

Supplementary Information

## Figures and Tables

**Table 1:** Characteristics and clinical outcomes of 1886 patients, by diagnosed infection, October 1, 2011 to September 30, 2012.

Characteristics	Diagnosed infection				
	Pneumonia	Sepsis	Urinary tract infection	Hepatobiliary or Intra-abdominal	Others
Total, N (%)	1213 (64.3)	215 (11.4)	182 (9.7)	147 (7.8)	129 (6.8)
*Demographic data*					
Age, mean (SD)	74.9 (14.5)	69.0 (15.9)	72.7 (16.7)	66.7 (17.2)	62.5 (16.0)
Males, N (%)	710 (58.5)	119 (55.3)	75 (41.2)	79 (53.7)	71 (55.0)
Ethnicity, N (%)					
Chinese	986 (81.3)	155 (72.1)	127 (69.8)	110 (74.8)	84 (65.1)
Malay	105 (8.7)	25 (11.6)	26 (14.3)	13 (8.8)	24 (18.6)
Indian	82 (6.8)	19 (8.8)	17 (9.3)	8 (5.4)	8 (6.2)
Other	40 (3.3)	16 (7.4)	12 (6.6)	16 (10.9)	13 (10.1)
Singapore residents, N (%)	1170 (96.5)	203 (94.4)	174 (95.6)	135 (91.8)	118 (91.5)
Private ward class, N (%)	104 (8.6)	20 (9.3)	16 (8.8)	20 (13.6)	12 (9.3)
*Medical history*					
Co-morbidities, N (%)					
Diabetes mellitus	384 (31.7)	69 (32.1)	72 (39.6)	41 (27.9)	44 (34.1)
Cardiovascular disease	237 (19.5)	40 (18.6)	24 (13.2)	16 (10.9)	22 (17.1)
Liver disease	32 (2.6)	10 (4.7)	9 (5.0)	16 (10.9)	1 (0.8)
Renal disease	241 (19.9)	52 (24.2)	48 (26.4)	22 (15.0)	26 (20.2)
Neoplasia	181 (14.9)	40 (18.6)	20 (11.0)	39 (26.5)	15 (11.6)
CNS disease	277 (22.8)	48 (22.3)	44 (24.2)	9 (6.1)	16 (12.4)
Chronic pulmonary disease	143 (11.8)	6 (2.8)	6 (3.3)	3 (2.0)	1 (0.8)
Charlson’s comorbidity index >5, N (%)	151 (12.4)	35 (16.3)	25 (13.7)	33 (22.4)	11 (8.5)
Prior hospitalisation, N (%)	478 (39.4)	90 (41.9)	90 (49.5)	52 (35.4)	41 (31.8)
Prior antibiotics, N (%)	939 (77.4)	166 (77.2)	156 (85.7)	111 (75.5)	104 (80.6)
Prior proton pump inhibitors, N (%)	721 (59.4)	143 (66.5)	138 (75.8)	92 (62.6)	79 (61.2)
*Current Admission*					
Length of stay prior to antibiotics, mean (SD)	8.9 (26.0)	8.7 (14.7)	11.1 (15.8)	5.5 (8.1)	14.9 (47.9)
Day of antibiotic prescription, N (%)					
Weekend or Public Holiday	357 (29.4)	46 (21.4)	60 (33.0)	40 (27.2)	33 (25.6)
Weekday	856 (70.6)	169 (78.6)	122 (67.0)	107 (72.8)	96 (74.4)
Time of antibiotic prescription, N (%)					
Night^a^	468 (38.6)	75 (34.9)	54 (29.7)	59 (40.1)	43 (33.3)
Day	745 (61.4)	140 (65.1)	128 (70.3)	88 (59.9)	86 (66.7)
Illness severity, N (%)					
C-reactive protein^b^ > 100mg/l	415 (38.4)	74 (37.9)	58 (34.5)	58 (51.3)	60 (52.6)
Leukocyte count <4 or >12 ×10^9/l	584 (48.1)	123 (57.2)	89 (48.9)	88 (59.9)	72 (55.8)
Serum creatinine^c^ >130μmol/l	293 (24.2)	81 (37.7)	49 (27.5)	40 (27.6)	43 (33.6)
ICU admission, N (%)	122 (10.1)	40 (18.6)	6 (3.3)	30 (20.4)	20 (15.5)
Prescribing physician, N (%)					
Senior	118 (9.7)	28 (13.0)	18 (9.9)	21 (14.3)	6 (4.7)
Junior	1095 (90.3)	187 (87.0)	164 (90.1)	126 (85.7)	123 (95.3)
Attending physician, N (%)					
Ethnic Chinese	887 (73.1)	148 (68.8)	141 (77.5)	109 (74.1)	97 (75.2)
Ethnic Indian	244 (20.1)	42 (19.5)	31 (17.0)	31 (21.1)	28 (21.7)
Other ethnicity	82 (6.8)	25 (11.6)	10 (5.5)	7 (4.8)	4 (3.1)
Clinical specialties, N (%)					
Medical	986 (81.3)	170 (79.1)	142 (78.0)	64 (43.5)	63 (48.8)
Surgical	227 (18.7)	45 (20.9)	40 (22.0)	83 (56.5)	66 (51.2)
Receipt of antibiotics according to ARUSC’s recommendations, N (%)	403 (33.2)	26 (12.1)	13 (7.1)	19 (12.9)	9 (7.0)
Clinical outcomes					
30-day all-cause mortality, N (%)	241 (19.9)	62 (28.8)	18 (9.9)	26 (17.7)	14 (10.9)
180-day *C. difficile* infection, N (%)	58 (4.8)	8 (3.7)	15 (8.2)	1 (0.7)	3 (2.3)
180-day MDRO infection, N (%)	69 (5.7)	12 (5.6)	25 (13.7)	13 (8.8)	21 (16.3)
	**Survivors at hospital discharge**				
Total Survivors, N (%)	953 (63.9)	144 (9.7)	163 (10.9)	119 (8.0)	113 (7.6)
30-day infection-related readmission	111 (11.6)	15 (10.4)	18 ((11.0)	6 (5.0)	17 (15.0)

^a^Night is defined as physician on-call hours from 1730 hours to 0730 hours.

^b^C-reactive protein closest to prescription date (within 7 days), missing in pneumonia (133/1213 = 11.0%), sepsis (20/215 = 9.3%), urinary tract infection (14/182 = 7.7%), hepatobiliary or intra-abdominal infection (34/147 = 23.1%), other infections (15/129 = 11.6%).

^c^Creatinine level closest to prescription date (within 7 days), missing in pneumonia (3/1213 = 0.2%), sepsis (0/215), urinary tract infection (4/182 = 2.2%), hepatobiliary or intra-abdominal infection (2/147 = 1.4%), other infections (1/129 = 0.8%).

**Table 2:**
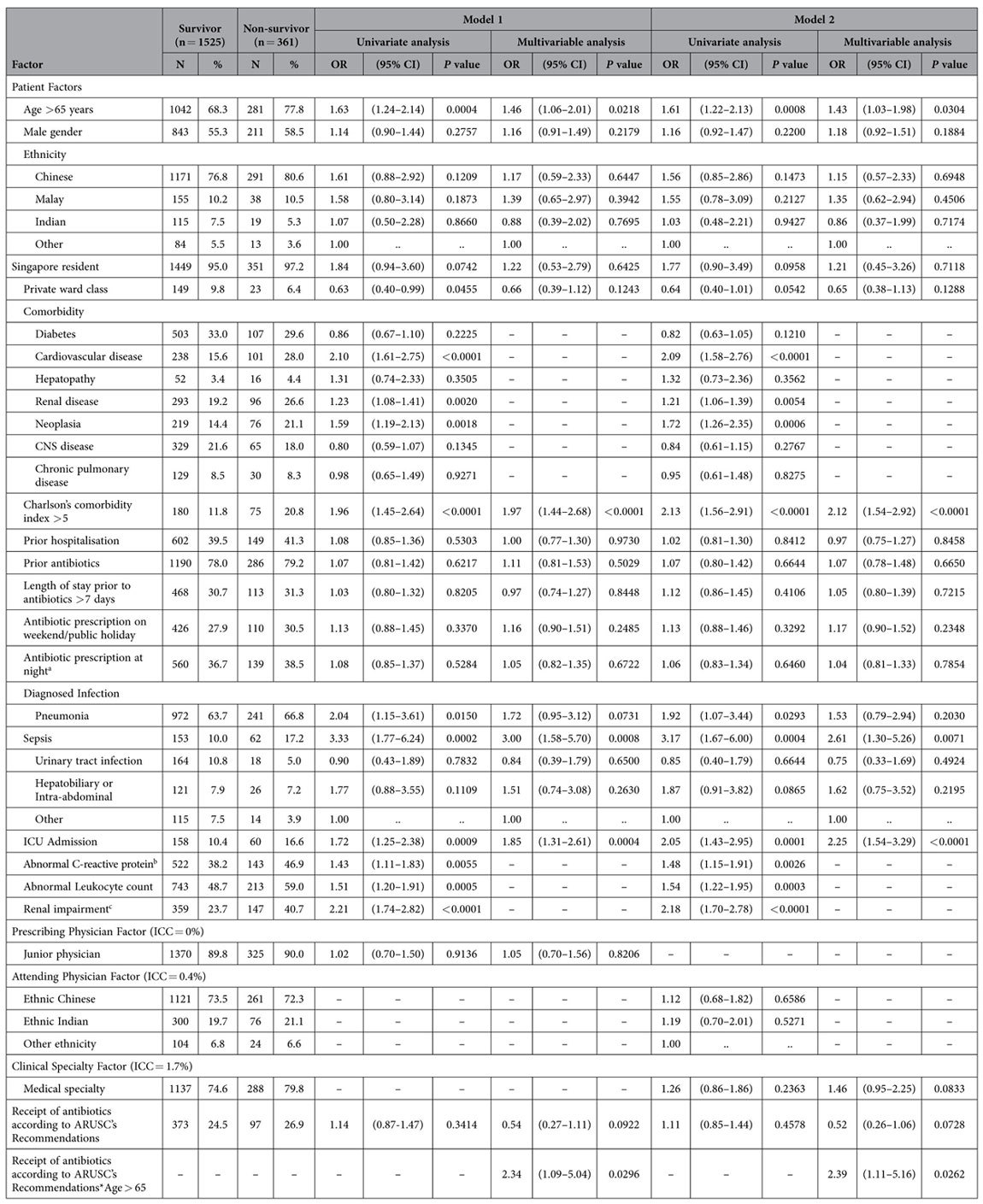
Results of univariate and multivariable analyses of factors associated with 30-day all-cause mortality.

(Model 1: 2-level logistic regression analysis of data on 1886 patients seen by 575 prescribing physicians; Model 2: 3-level logistic regression analysis of data on 1886 patients seen by 220 attending physicians in 19 clinical specialties). Abbreviations: CNS, central nervous system; ICC, intraclass correlation coefficient; ICU, intensive care unit; OR, odds ratio; CI, confidence interval.

^a^Night is defined as physician on-call hours from 1730 hours to 0730 hours.

^b^C-reactive protein closest to prescription date (within 7 days), missing in survivors (160/1525 = 10.5%) and non-survivors (56/361 = 15.5%).

^c^Creatinine level >130μmol/l within 7 days of antibiotic prescription, missing in survivors (10/1525 = 0.7%) and non-survivors (0/361).

**Table 3:** Propensity score (PS)-adjusted and conventional multivariable analyses of factors associated with 30-day all-cause mortality.

Factor	Model 1						Model 2					
	PS^a^-adjusted multivariable analysis			Conventional multivariable analysis			PS^a^-adjusted multivariable analysis			Conventional multivariable analysis		
	OR	(95% CI)	*P* value	OR	(95% CI)	*P* value	OR	(95% CI)	*P* value	OR	(95% CI)	*P* value
Patient Factors												
Age >65 years	**1.46**	**(1.07–2.00)**	**0.0179**	**1.46**	**(1.06–2.01)**	**0.0218**	**1.41**	**(1.02–1.95)**	**0.0353**	**1.43**	**(1.03–1.98)**	**0.0304**
Male gender	1.19	(0.93**–**1.51)	0.1616	1.16	(0.91**–**1.49)	0.2179	1.19	(0.94**–**1.52)	0.1540	1.18	(0.92**–**1.51)	0.1884
Ethnicity												
Chinese	1.13	(0.58**–**2.22)	0.7164	1.17	(0.59**–**2.33)	0.6447	1.10	(0.55**–**2.17)	0.7890	1.15	(0.57**–**2.33)	0.6948
Malay	1.31	(0.62**–**2.76)	0.4811	1.39	(0.65**–**2.97)	0.3942	1.27	(0.60-2.71)	0.5376	1.35	(0.62**–**2.94)	0.4506
Indian	0.86	(0.38**–**1.95)	0.7187	0.88	(0.39**–**2.02)	0.7695	0.82	(0.36**–**1.87)	0.6354	0.86	(0.37**–**1.99)	0.7174
Other	1.00	..	..	1.00	..	..	1.00	..	..	1.00	..	..
Singapore resident	1.23	(0.54**–**2.80)	0.6170	1.22	(0.53**–**2.79)	0.6425	1.23	(0.54**–**2.81)	0.6252	1.21	(0.45**–**3.26)	0.7118
Private ward class	0.68	(0.40**–**1.13)	0.1386	0.66	(0.39**–**1.12)	0.1243	0.67	(0.39**–**1.12)	0.1269	0.65	(0.38**–**1.13)	0.1288
Charlson’s comorbidity index >5	**2.00**	**(1.47–2.71)**	**<0.0001**	**1.97**	**(1.44–2.68)**	**<0.0001**	**2.18**	**(1.59–2.99)**	**<0.0001**	**2.12**	**(1.54–2.92)**	**<0.0001**
Prior hospitalisation	–	–	–	1.00	(0.77**–**1.30)	0.9730	–	–	–	0.97	(0.75**–**1.27)	0.8458
Prior antibiotics	–	–	–	1.11	(0.81**–**1.53)	0.5029	–	–	–	1.07	(0.78**–**1.48)	0.6650
Length of stay prior to antibiotics >7 days	–	–	–	0.97	(0.74**–**1.27)	0.8448	–	–	–	1.05	(0.80**–**1.39)	0.7215
Antibiotic prescription on weekend/public holiday	–	–	–	1.16	(0.90**–**1.51)	0.2485	–	–	–	1.17	(0.90**–**1.52)	0.2348
Antibiotic prescription at night^b^	–	–	–	1.05	(0.82**–**1.35)	0.6722	–	–	–	1.04	(0.81**–**1.33)	0.7854
Diagnosed Infection												
Pneumonia	–	–	–	1.72	(0.95**–**3.12)	0.0731	–	–	–	1.53	(0.79**–**2.94)	0.2030
Sepsis	–	–	–	**3.00**	**(1.58–5.70)**	**0.0008**	–	–	–	**2.61**	**(1.30–5.26)**	**0.0071**
Urinary tract infection	–	–	–	0.84	(0.39**–**1.79)	0.6500	–	–	-	0.75	(0.33**–**1.69)	0.4924
Hepatobiliary or Intra-abdominal	–	–	–	1.51	(0.74**–**3.08)	0.2630	–	–	–	1.62	(0.75**–**3.52)	0.2195
Other	–	–	–	1.00	..	..	–	–	–	1.00	..	..
ICU Admission	**1.96**	**(1.40–2.75)**	**<0.0001**	**1.85**	**(1.31–2.61)**	**0.0004**	**2.47**	**(1.70–3.58)**	**<0.0001**	**2.25**	**(1.54–3.29)**	**<0.0001**
Prescribing Physician Factor (ICC = 0% [PS], 0% [Conventional])												
Junior physician	1.02	(0.69**–**1.51)	0.9340	1.05	(0.70**–**1.56)	0.8206	–	–	–	–	–	–
Attending Physician Factor (ICC = 0.1% [PS], 0% [Conventional])												
Clinical Specialty Factor (ICC = 0.8% [PS], 1.0% [Conventional])												
Medical specialty	–	–	–	–	–	–	**1.53**	**(1.02–2.30)**	**0.0413**	1.46	(0.95**–**2.25)	0.0833
Receipt of antibiotics according to ARUSC’s Recommendations	0.54	(0.26**–**1.10)	0.0882	0.54	(0.27**–**1.11)	0.0922	0.52	(0.25**–**1.05)	0.0686	0.52	(0.26**–**1.06)	0.0728
Receipt of antibiotics according to ARUSC’s Recommendations*Age > 65	**2.32**	**(1.08**–**4.98)**	**0.0302**	**2.34**	**(1.09–5.04)**	**0.0296**	**2.38**	**(1.11–5.13)**	**0.0267**	**2.39**	**(1.11–5.16)**	**0.0262**

(Model 1: 2-level logistic regression analysis of data on 1886 patients seen by 575 prescribing physicians; Model 2: 3-level logistic regression analysis of data on 1886 patients seen by 220 attending physicians in 19 clinical specialties). Abbreviations: CNS, central nervous system; ICC, intraclass correlation coefficient; ICU, intensive care unit; OR, odds ratio; CI, confidence interval.

^a^Propensity score derived from diagnosed infection, time and day of antibiotic prescription, hospitalisation days prior to antibiotics, prior hospitalisation, and prior antibiotics.

^b^Night is defined as physician on-call hours from 1730 hours to 0730 hours.

**Table 4:** Association between receipt of antibiotics according to ARUSC’s recommendations and 30-day all-cause mortality risk, according to age group, October 1, 2011 to September 30, 2012.

Analysis and receipt of antibiotics according to ARUSC’s recommendations	Age < = 65 years		Age > 65 years		*P*-interaction^a^
	OR	(95% CI)	OR	(95% CI)	
Unadjusted analysis					
Non-receipt	1.00	Referent	1.00	Referent	0.0187
Receipt	0.52	(0.26–1.05)	1.29	(0.97–1.72)	
Adjusted analysis^b^					
Non-receipt	1.00	Referent	1.00	Referent	0.0302
Receipt	0.45	(0.20–1.00)	1.28	(0.91–1.82)	

Abbreviations: OR, odds ratio; CI, confidence interval.

^a^Multiplicative scale.

^b^Adjusted using a propensity score derived from diagnosed infection, time and day of antibiotic prescription, hospitalisation days prior to antibiotics, prior hospitalisation, and prior antibiotics, and further adjusted for prescribing physician’s seniority, and patient’s gender, ethnicity, resident status, ward class, Charlson’s comorbidity index >5, and ICU admission.
