# Supplementary material for: Mortality Benefits of Antibiotic Computerised Decision Support System: Modifying Effects of Age
Source: Sci Rep. 2015 Nov 30;5:17346. doi: 10.1038/srep17346 (PMC4663624; doi:10.1038/srep17346)
Supplement: Supplementary Information [file srep17346-s1.pdf]

SUPPLEMENT

Mortality Benefits of Antibiotic Computerised Decision Support System:

Modifying Effects of Age

Angela LP Chow, PhD [1, 2], David C Lye, FRACP [3, 4], Onyebuchi A Arah, PhD [2, 5]

[1] Department of Clinical Epidemiology, Institute of Infectious Disease and Epidemiology, Tan Tock Seng Hospital, Singapore

[2] Department of Epidemiology, Fielding School of Public Health, University of California, Los Angeles (UCLA), Los Angeles, United States

[3] Department of Infectious Diseases, Institute of Infectious Disease and Epidemiology, Tan Tock Seng Hospital, Singapore

[4] Yong Loo Lin School of Medicine, National University of Singapore, Singapore

[5] Center for Health Policy Research, University of California, Los Angeles (UCLA), Los Angeles, United States

Supplementary Table S1. Empiric Antibiotics Recommended by Computerised Decision Support System, by Commonly Diagnosed Infection

Diagnosed infection	Recommended antibiotics for empiric therapy
Community-acquired pneumonia	Amoxicillin-clavulanate, Clarithromycin
Severe community-acquired pneumonia	Penicillin, Ceftazidime, Azithromycin
Healthcare-associated pneumonia	Piperacillin-tazobactam, Vancomycin
Acute cholecystitis/cholangitis/ gallbladder empyema	Amoxicillin-clavulanate, Gentamicin
Liver abscess	Amoxicillin-clavulanate
Pyelonephritis/ perinephric abscess	Cefazolin, Gentamicin
Cystitis	Co-trimoxazole
Sepsis	Cloxacillin, Ceftazidime, Clindamycin
